# Chemical Constituents and an Alternative Medicinal Veterinary Herbal Soap Made from *Senna macranthera*

**DOI:** 10.1155/2015/217598

**Published:** 2015-03-03

**Authors:** Flávia Inoue Andrade, Gislaine Aparecida Purgato, Thalita de Faria Maia, Raoni Pais Siqueira, Sâmia Lima, Gaspar Diaz, Marisa Alves Nogueira Diaz

**Affiliations:** ^1^Departamento de Bioquímica e Biologia Molecular, Universidade Federal de Viçosa, 36570-000 Viçosa, MG, Brazil; ^2^Departamento de Química, Universidade Federal de Minas Gerais, 31270-901 Belo Horizonte, MG, Brazil

## Abstract

Upon undergoing biomonitoring, the most active dichloromethane extract retrieved from *Senna macranthera* roots led to the isolation of three main compounds: emodine, physione, and chrysophanol. In this sequence, these compounds revealed a potential antibacterial activity against *Staphylococcus aureus* strains isolated from animals with mastitis infections with minimum inhibitory concentration (MIC) values of 20, 90, and 90 *μ*g mL^−1^, respectively. Therefore, an herbal soap was also produced from this same active extract. This soap was tested *in vitro* using gloves contaminated by animals with bovine mastitis that had been discarded after use by milkers and showed similar results to previously tested compounds. These results indicate the potential of this plant as an alternative veterinary medicine for the production of antibacterial soaps that aimed at controlling bovine mastitis infections in small Brazilian farms.

## 1. Introduction

In Brazil, the use of medicinal plants contributes significantly to primary health care. These vegetation species are used in the form of crude extracts, infusions, or plasters to treat common infections in humans and animals [1]. The alternative use of medicinal plants to treat common infections in animals is increasing, as is knowledge in ethnoveterinary studies, especially in some regions of developing countries that have limited access to conventional medicines for animal health care, as they are often unaffordable to poor rural farmers [2].

Plants are rich in a wide variety of secondary metabolites, such as tannins, terpenoids, alkaloids, anthraquinones, and flavonoids, among other compounds, which have demonstrated* in vitro* antimicrobial properties. Of this wide variety of secondary metabolites, the anthraquinones have proven to be outstanding due to their broader action against several microorganisms [3]. These compounds are mainly found in* Senna macranthera* (Collad. Irwin et Barn) species, also known, by Brazilian local farmers, as* pau fava* and* fedegoso*, which belongs to the Fabaceae family. It is a native plant from Brazil that is commonly found in forests and is used as an ornamental tree. However, the* Senna* genus is also widely distributed in tropical and subtropical regions throughout the world, and it has been extensively, chemically, and pharmacologically investigated [4]. Folk medicine in Brazil has made use of these species (*Fedegoso* and* Senna*) as a remedy for various diseases, especially to treat infectious diseases [2]. The aerial parts and roots of* S. macranthera* have been used by some farmers to treat bovine mastitis, a chronic infection that produces an inflammatory response in cow's udders and that is caused by* Staphylococcus aureus*, the main etiologic causative agent of this disease.* Staphylococci* cause chronic infections due to their ability to adhere to many types of surfaces, in turn developing a matrix-encased community of cells, called biofilms, which hinders antibiotic action [5]. Therefore, the search for new antibiotics for the treatment of bovine mastitis must focus on inhibiting biofilm formation by plants or active compounds. Thus, this work aimed to test the bioassay-guided isolation compounds responsible for antibacterial activity and to check the effect of plant extracts on biofilm formation. This study also sought to produce an herbal soap from the active extract of* S. macranthera* and assess its antiseptic potential for alternative uses in veterinary medicine, especially with regard to the treatment of bovine mastitis at small farms in rural areas in Brazil.

## 2. Materials and Methods

### 2.1. General

Silica gel (70–230 mesh) and glass columns were used for column chromatography. All of the solvents used were of analytical grade. The melting point was determined using a Thermopan apparatus (C. Reichest Optische Wercke A G). ^1^H and ^13^C nuclear magnetic resonance (NMR) spectra were recorded on 300 MHz and 75 MHz NMR spectrometers, respectively, (Mercury 300 spectrometer). Tetra methylsilane (TMS) was used as an internal standard. The IR spectra were measured in a Perkin Elmer Paragon 1000 FTIR spectrophotometer, using potassium bromide (1% w/w) scanning from 400 to 4000 cm^−1^.

### 2.2. Plant Material

*S. macranthera* was collected from Violeira, a neighborhood located (20°44′5′′S, 40°51′26′′W) in Viçosa, MG, Brazil, in February 2011, and an authenticated voucher (VIC 39644) was deposited in the university's herbarium. The roots were dried at 40°C in an air circulation oven. Dried roots (800 g) were extracted using* n*-hexane, dichloromethane, and ethanol for 5 days each and repeated at least five times. The solvents were concentrated under reduced pressure until they were completely dry, and the extracts were stored at 4°C.

### 2.3. Phytochemical Studies

**Figure 1:**
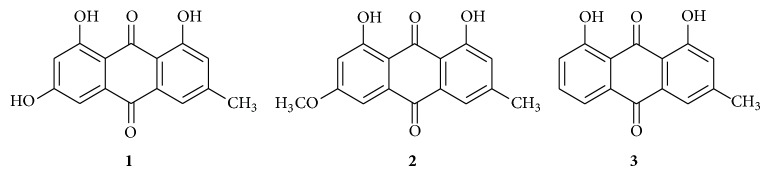
Isolated compounds from the most active fraction of the* Senna macranthera* roots.

The active dichloromethane extract from the* S. macranthera* roots was first subjected to a chromatography column, using dichloromethane as the eluent. The eluent polarity was then gradually increased by adding ethyl acetate, yielding 50 fractions. The fractions obtained were subsequently submitted to biological assay using the* S. aureus* strain 3828 (identified by the Embrapa Dairy Cattle from the Milk Microbiology Laboratory) as an infectious reference microorganism. The bioassayed positive fractions were submitted to a preparative thin layer chromatography (PTLC) eluted with dichloromethane/EtOAc (8 : 2), allowing for the isolation of three compounds:** 1** (6 mg),** 2** (4 mg), and** 3** (5 mg) (Figure 1).

### 2.4. Bacterial Strains and Culture Media

The bacterial strains used in this study, which were isolated from animals with mastitis infections, were kindly provided by the Embrapa Dairy Cattle from the Milk Microbiology Laboratory, (Juiz de Fora, MG, Brazil). Six* Staphylococcus aureus* strains (3828, 3893, 4075, 4125, 4158, and 4182) and* Streptococcus agalactiae* (3849),* Streptococcus bovis* (550), and* Escherichia coli* (24) bacteria (one strain of each) were used to determine the antimicrobial activity of the extracts, fractions, and isolated compounds. Bacteria were routinely cultured on brain heart infusion (BHI) agar at 37°C for 16 h before conducting the experiments. The cell concentration was adjusted to 10^6^ CFU mL^−1^ with an optical density set at 600 nm. Stock cultures were maintained in BHI agar containing 25% glycerol at −80°C.

### 2.5. Antibacterial Screening Assay

Hole-plate diffusion assay was initially performed to test the antibacterial activity of the fractions obtained from crude extracts of the roots. To accomplish this, the bacteria were cultivated overnight, and a suspension containing 10^6^ CFU mL^−1^ was spread on plates containing Müeller-Hinton agar (Himedia). Holes of approximately 5 × 3 mm were made in the agar and filled with 30 *μ*L of the fraction stock solutions (50 mg mL^−1^) and 10 *μ*g mL^−1^ for compounds** 1**,** 2**, and** 3**. After incubation at 37°C for 24 h, inhibition zones were measured in millimeters and compared to the controls. The antibiotic ciclopirox olamine (Uci-Farma) was used as the positive control due to its antibacterial properties [6]. Dimethylsulfoxide (DMSO) was used as a negative control. Tests were performed twice in triplicate. The minimum inhibitory concentration (MIC) of compounds** 1**,** 2**, and** 3** was determined by applying a broth microdilution method followed by incubation at 37°C for 24 h and by observing media turbidity. Tests were performed twice in triplicate.

### 2.6. Effect of Plant Extracts on Cell Adherence

The effect of subinhibitory concentrations of active dichloromethane extract established biofilms was evaluated according to Nostro et al. [7] with few modifications. Bacterial suspensions were inoculated on microplates containing 180 *μ*L of BHI until reaching the final concentration of 10^6^ CFU mL^−1^ and incubated at 37°C for 24 h. The supernatant was withdrawn; wells were washed three times with 0.85% saline and filled again with BHI containing different concentrations of the active extracts (MIC, 1/2 MIC, 1/4 MIC, 1/8 MIC, and 1/16 MIC), followed by incubation at 37°C for 24 h. The biofilm inhibitory concentration (BIC) was defined as the concentration at which no visible microbial growth was observed. The assay was performed twice in triplicate.

### 2.7. Effect of Plant Extracts on Biofilm Formation

Bacterial suspensions were inoculated on microplates containing 180 *μ*L of BHI with different concentrations of the active extracts (MIC, 1/2 MIC, 1/4 MIC, 1/8 MIC, and 1/16 MIC) until reaching the final concentration of 10^6^ CFU mL^−1^ and incubated at 37°C for 24 h. The supernatant was withdrawn and wells were washed three times with 0.85% saline. The remaining bacterial mass was dried at 37°C for 15 min and stained with 200 *μ*L of crystal violet 0.1% for 30 min. Wells were rewashed and dried as previously described, followed by the addition of 300 *μ*L of ethanol and the measurement of absorbance at 630 nm. The test was performed twice in triplicate.

### 2.8. Production of Herbal Soap

The active extract of* S. macranthera* (250 mg) was incorporated into a soap produced according to its patent: 1005633-5 [8]. Later, the semi-solid mixture was poured into a mold and allowed to solidify. Soap without the extract was also produced to be used as a reference product.

### 2.9. Antibacterial Assay of the Herbal Soap

The agar-dilution method was employed in an* in vitro* evaluation. The herbal soap (1.0 g) was dissolved in distilled water (50 mL) to obtain a 2% suspension. The suspension was vigorously shaken to dissolve the soap, to disperse the foam, and to homogenize the suspension. Next, 1.0 mL of the soap solution was added to 20 mL of sterile molten culture media in Petri dishes and allowed to set. One hundred *μ*L of suspension containing 10^6^ CFU mL^−1^ of a resistant 3828* S. aureus* strain was then streaked on the plates. After incubation at 37°C for 24 h, inhibition zones were compared to the control to observe the presence or absence of microbial growth.

Gloves contaminated with* S. aureus* from animals with mastitis infection were used to perform the* in vivo* evaluation (topical test according to our institutional ethical protocol number 773.182). The herbal soap (1.0 g) was dissolved in distilled water (100 mL) to obtain a 1% suspension. This suspension was then vigorously shaken to dissolve the soap, to disperse the foam, and to homogenize it. Thereafter, the gloves (12 pairs, 6 for each control soap and herbal soap treatment) were immersed in these solutions for 30 minutes. Before being immersed in the soap, the gloves that the milkers had used to milk the cow's udder, which had been contaminated with* S. aureus*, were swabbed, and the sample was placed in bottles with sterile normal saline. After being immersed in both the herbal and control soaps, the gloves were again swabbed, and the samples were placed in separate bottles with normal sterile saline solution. Aliquots from the respective treatments were cultured on an agar plate at 37°C for 24 h to observe the presence or absence of microbial growth.

## 3. Results and Discussion

Purification of the most active fraction obtained from the bioassay-guided active dichloromethane extract from* S. macranthera* roots led to the isolation of three active compounds, which were characterized as emodine (**1**), physcione (**2**), and chrysophanol (**3**) (Figure 1).

*Compound* ***1***. It is an orange amorphous powder from CHCl_3_, mp 261–264°C. The IR spectrum showed absorptions referring to a hydroxyl group (3382 cm^−1^) and carbonilic groups (1677 and 1627 cm^−1^, resp.). The EI mass spectrum of** 1** displayed a molecular ion [M]^+^ at* m/z* 270 (100%), which is in accordance with the molecular formula C_15_H_10_O_5_. Other major fragments were observed at* m/z* 269 and 241. ^1^H NMR (300 MHz, CDCl_3_): *δ* 2.42 (3H, s), 6.55 (1H, d,* J* = 1.0 Hz, H-2), 7.08 (1H, d,* J* = 1.0 Hz, H-4), 7.17 (1H, d,* J* = 2.4, H-7), 7.55 (1H, d,* J* = 2.4 Hz, H-5), 12.08 (1H, s, H-1), 12.28 (1H, s, H-8); ^13^C NMR (75 MHz, CDCl_3_): *δ* 21.2 (C-2′), 106.7 (C-5), 108.9 (C-7), 162.2 (C-1), 167.3 (C-6), 184.3 (C-10), 192.3 (C-9).

*Compound* ***2***. It is an orange amorphous powder from CDCl_3_, mp 206–209°C. The IR spectrum showed absorptions referring to a hydroxyl group (3340 cm^−1^) and carbonilic groups (1656 and 1619 cm^−1^, resp.). The EI mass spectrum of** 2** displayed a molecular ion [M]^+^ at* m/z* 284 (100%), which is in accordance with the molecular formula C_16_H_12_O_5_. Other major fragments were also observed at* m/z* 269 and 240. ^1^H NMR (300 MHz, CDCl_3_): *δ* 2.42 (3H, s, CH_3_), 3.93 (3H, s, OCH_3_), 6.48 (1H, s, H-2), 6.58 (1H, s, H-7), 6.92 (1H, s, H-4), 7.12 (1H, s, H-5), 12.08 (1H, s, H-1), 12.28 (1H, s, H-8); ^13^C NMR (75 MHz, CDCl_3_): *δ* 21.2 (C-2′), 55.7 (C-3′), 106.7 (C-5), 108.9 (C-7), 162.2 (C-1), 167.3 (C-6), 184.3 (C-10), 190.8 (C-9).

*Compound* ***3***. It is a white amorphous powder from CDCl_3_, mp 193-194°C. The IR spectrum showed absorptions referring to a hydroxyl group (3340 cm^−1^) and carbonilic groups (1656 and 1619 cm^−1^, resp.). The EI mass spectrum of** 3** displayed a molecular ion [M]^+^ at* m/z* 254 (100%), which is in accordance with the molecular formula C_15_H_10_O_5_. Other major fragments were also observed at* m/z* 212, 197, 169, and 43. ^1^H NMR (300 MHz, CDCl_3_): *δ* 2.47 (3H, s, CH_3_), 7.10 (1H, d,* J* = 1.1 Hz, H-2), 7.31 (1H, dd,* J* = 7.62, 1.4 Hz, H-7), 7.67 (1H, d,* J* = 1.1 Hz, H-4), 7.70 (1 H, t,* J* = 8.0 Hz, H-6), 7.85 (1H, dd,* J* = 7.52, 1.4 Hz, H-5), 12.03 (1H, s, H-1), 12.14 (1H, s, H-8); ^13^C NMR (75 MHz, CDCl_3_): *δ* 22.4 (C-2′), 113.7 (C-13), 115.9 (C-12), 119.9 (C-5), 121.4 (C-4), 121.4 (C-7), 124.5 (C-2), 133.3 (C-14), 133.6 (C-11), 136.9 (C-6), 149.3 (C-3), 162.4 (C-1), 162.7 (C-8), 182.0 (C-10), 192.5 (C-9).

All three isolated compounds emodine (**1**), physcione (**2**), and chrysophanol (**3**) are well-known and were identified. In addition, these compounds also underwent spectroscopic analysis to compare the reported spectroscopic data. These compounds proved to be active against Gram-positive and Gram-negative microorganisms [9, 10], are very common in the genus* Senna*, and are known to have several biological activities, among them, remarkable antimicrobial activity [11]. The compounds were tested against* S. aureus* isolated strains, the main causative agent of bovine mastitis, and the obtained results are presented in Table 1.

Results from Table 1 are in accordance with the information provided by local farmers that use this plant to treat bovine mastitis. The three isolated compounds showed good to excellent antimicrobial activities, ranging from 20 to 190 *μ*g mL^−1^, and are most likely responsible for the antimicrobial activity observed with the tested dichloromethane extract.

Similarly, as can be seen in Table 2, both dichloromethane and ethanol extracts showed good activity in combatting* S. aureus*, the main causative agent of bovine mastitis. Likewise, the same extracts also showed positive activity against other microorganisms, such as* Streptococcus agalactiae*,* Streptococcus bovis*, and* Escherichia coli* (Table 3), which are also causative agents of this disease.

According to Table 3, the dichloromethane extract can be considered more active than an ethanol extract against the tested bacteria, except for* E. coli*, whose activity was lower.

As the two extracts showed significant antibacterial activity against the three tested bacteria, the MIC have been made with all of them (Table 4). The attained MIC values of the most active dichloromethane extract against the tested bacteria proved to be better when compared to that previously found from other extracts of plants with antimicrobial activities [12, 13]. According to Aligiannis criteria [14], the dichloromethane extract can be considered strong inhibitors for* S. aureus* and* S. agalactiae* and moderate for* S. bovis*. Hence, the ethanol extract can be considered moderate for the three tested microorganisms (Table 4).

Several studies have shown that biofilm formation is a key factor in the establishment and persistence of infections caused by* S. aureus* and* S. agalactiae* in animals with mastitis infection [15]. Currently, no vaccines or immunotherapies have been approved to treat these* Staphylococcal* infections. This area of research has peaked considerable interest, mainly for the use of small molecules to combat* staphylococcal* biofilms [16]. Thus, the dichloromethane and ethanol extracts were tested in subinhibitory concentrations of MIC values (1/2, 1/4, 1/8, and 1/16) to evaluate BIC and adhesion on preformed biofilms against these microorganisms. The results showed that the dichloromethane extract presented the lowest BIC values for* S. aureus *and* S. agalactiae*, equal to 1/8 and 1/2, respectively, of the obtained MIC values. For the ethanol extract, the BIC values were 1/4 and 1/2 on* S. aureus* and* S. agalactiae*, respectively (Table 5).

According to Table 5, the best BIC value (0.063 mg mL^−1^) obtained for the dichloromethane extract against* S. aureus* can indicate that this extract can inhibit bacterial film formation in the initial phase of adhesion and formation of biofilms. This value was similar to those found for antibiotic substances reported in prior literature [8].

Due to the promising results concerning the antibacterial activities of active extracts from* S. macranthera* roots and compounds isolated through biomonitoring, according to the above discussion, the authors of this study were encouraged to prepare an herbal soap containing the active extract. As a result, the herbal soaps should represent a new alternative that is easily accessible for use by farmers from small farms in the rural areas of Brazil who already use this plant in an attempt to control bovine mastitis. For this purpose, a new procedure was devised for antibacterial tests. The herbal soap was tested* in vivo,* using gloves that had been contaminated by animals with bovine mastitis and that had been discarded after having been used by the milkers of the university's bovine culture sector.

**Figure 2:**
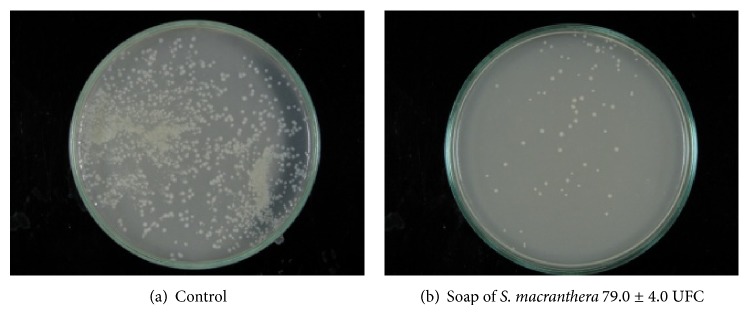
*In vitro* antibacterial activities of herbal soap produced with the most active extract of* S. macranthera.* Tests were performed in triplicate.

Figure 2 showed that the herbal soap obtained from the most active dichloromethane extract of the* S. macranthera* roots reduces the bacterial load to 79.0 ± 4.0 CFU. This result is in agreement with those observed by small farmers.

**Figure 3:**
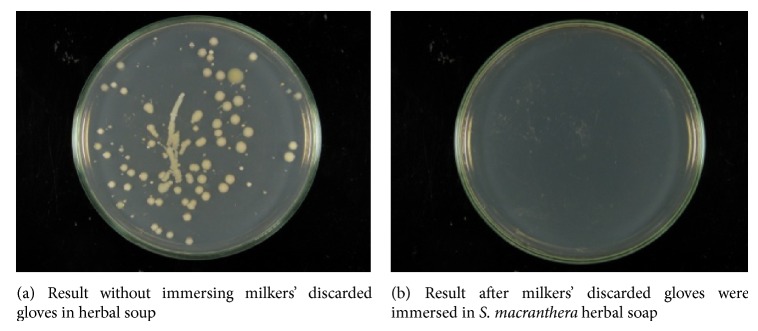
*In vivo* antibacterial activities of herbal soap with the active extract of* Senna macranthera* and the milkers' discarded gloves.

Figure 3 shows the results observed in the* in vivo* evaluation with the milkers' discarded gloves immersed or not in the 1% suspension of herbal soap together with an active extract of* S. macranthera*.  Figure 3(a) showed the microbial growth in the Petri dishes in which the gloves were not immersed in the herbal soap suspension. By contrast, Figure 3(b) showed no microbial growth in the Petri dishes after the milkers' discarded gloves had been immersed for 30 min in the herbal soap suspension.

The results obtained from* in vivo* tests (Figure 3) suggested that the active extract of this plant can be incorporated into herbal soaps that aimed at cleaning the animals' udders before milking, especially in small farms.

## 4. Conclusion

In summary, the inhibitory effects of the active extract of* S. macranthera* against* S. aureus *strains can be attributed to anthraquinone compounds** 1**,** 2**, and** 3**, found in the roots of this plant. The activity of the dichloromethane extract may well be associated with the synergism among the isolated compounds and other compounds that have not yet been isolated from this plant. Nevertheless, the herbal soap produced with an active extract of* S. macranthera* demonstrated a high inhibition against* S. aureus*, when the milkers' discarded gloves were immersed in the 1% suspension of herbal soap, indicating the potential of this plant as an excipient in the production and use of antiseptic soaps to control bovine mastitis infections, especially in small farms.

## Figures and Tables

**Table 1:** MIC (*μ*g mL^−1^) values of compounds isolated from a dichloromethane extract of *S. macranthera* roots against *S. aureus* strains.

Microorganisms	Compounds			
	Emodine (1)	Physcione (2)	Chrysophanol (3)	Ciclopirox olamine
*S. aureus* 3828	60	120	190	50
*S. aureus* 4125	20	90	90	50
*S. aureus* 4158	40	100	190	50

**Table 2:** Antibacterial activity of the dichloromethane and ethanol extracts from *S. macranthera* roots against *Staphylococcus aureus* strains.

*S. aureus* strains	Dichloromethane	Ethanol	Ciclopirox olamine	DMSO
Inhibition zones (mm ± SD)				
3828	16.0 ± 0.58	10.0 ± 0.58	17.0 ± 0.71	0.00
3893	13.0 ± 0.48	12.0 ± 0.47	18.0 ± 0.58	0.00
4075	15.0 ± 0.28	10.0 ± 0.25	15.0 ± 0.45	0.00
4125	16.0 ± 0.16	15.0 ± 0.23	16.0 ± 0.35	0.00
4158	15.0 ± 0.35	7.0 ± 0.18	16.0 ± 0.58	0.00
4182	10.0 ± 0.58	8.0 ± 0.15	14.0 ± 0.45	0.00

**Table 3:** Antibacterial activity of the dichloromethane and ethanol extracts against *Streptococcus agalactiae* (3849), *Streptococcus bovis* (550), and *Escherichia coli* (24) strains.

Extracts	Microorganisms		
	*S. agalactiae* (3849)	*S. bovis* (550)	*E. coli* (24)
	Inhibition zones ± SD (mm)		
Dichloromethane	15.0 ± 0.28	8.0 ± 0.20	1.3 ± 0.30
Ethanol	10.0 ± 0.52	4.0 ± 0.36	2.0 ± 0.28
Ciclopirox olamine^*^	15.0 ± 0.38	18.0 ± 0.20	18.0 ± 0.47
DMSO^**^	0.00	0.00	0.00

^*^Positive control. ^**^Negative control.

**Table 4:** MIC (mg mL^−1^) values of activity extracts from *S. macranthera* roots against *S. aureus*, *S. agalactiae*, and *S. bovis*.

Microorganisms	Extract	MIC
*S. aureus* (3828)	Dichloromethane	0.5
	Ethanol	1.0
	Positive control	0.05

*S. agalactiae* (3849)	Dichloromethane	0.6
	Ethanol	0.8
	Positive control	0.05

*S. bovis* (550)	Dichloromethane	0.8
	Ethanol	1.0
	Positive control	0.05

**Table 5:** BIC (mg mL^−1^) values of the dichloromethane and ethanol extracts against *S. aureus *and *S. agalactiae* bacteria.

Extracts	Microorganisms	BIC
Dichloromethane	*S. aureus* (*3828*)	0.063
	*S. agalactiae* (3849)	0.3
	Positive control	0.025

Ethanol	*S. aureus* (3828)	0.25
	*S. agalactiae* (3849)	0.4
	Positive control	0.025
